# Quantifying the Interactions between Maternal and Fetal Heart Rates by Transfer Entropy

**DOI:** 10.1371/journal.pone.0145672

**Published:** 2015-12-23

**Authors:** Faezeh Marzbanrad, Yoshitaka Kimura, Marimuthu Palaniswami, Ahsan H. Khandoker

**Affiliations:** 1 Electrical and Electronic Engineering Department, University of Melbourne, Melbourne, VIC 3010, Australia; 2 Graduate School of Medicine, Tohoku University, Sendai, Japan; 3 Biomedical Engineering Department, Khalifa University of Science, Technology and Research, Abu Dhabi, UAE; Université de Montréal, CANADA

## Abstract

Evidence of the short term relationship between maternal and fetal heart rates has been found in previous studies. However there is still limited knowledge about underlying mechanisms and patterns of the coupling throughout gestation. In this study, Transfer Entropy (TE) was used to quantify directed interactions between maternal and fetal heart rates at various time delays and gestational ages. Experimental results using maternal and fetal electrocardiograms showed significant coupling for 63 out of 65 fetuses, by statistically validating against surrogate pairs. Analysis of TE showed a decrease in transfer of information from fetus to the mother with gestational age, alongside the maturation of the fetus. On the other hand, maternal to fetal TE was significantly greater in mid (26–31 weeks) and late (32–41 weeks) gestation compared to early (16–25 weeks) gestation (Mann Whitney Wilcoxon (MWW) p<0.05). TE further increased from mid to late, for the fetuses with RMSSD of fetal heart rate being larger than 4 msec in the late gestation. This difference was not observed for the fetuses with smaller RMSSD, which could be associated with the quiet sleep state. Delay in the information transfer from mother to fetus significantly decreased (p = 0.03) from mid to late gestation, implying a decrease in fetal response time. These changes occur concomitant with the maturation of the fetal sensory and autonomic nervous systems with advancing gestational age. The effect of maternal respiratory rate derived from maternal ECG was also investigated and no significant relationship was found between breathing rate and TE at any lag. In conclusion, the application of TE with delays revealed detailed information on the fetal-maternal heart rate coupling strength and latency throughout gestation, which could provide novel clinical markers of fetal development and well-being.

## 1 Introduction

Monitoring of Fetal Heart Rate (FHR) has been widely used for a reliable assessment of fetal wellbeing and development. In particular, acquisition of FHR through non-invasive fetal electrocardiogram (fECG), even in its current stage of development, provides an accurate estimation of FHR and its beat-to-beat variability [1–3]. FHR is influenced by not only the fetal conditions including behaviorial state and maturation, but also the maternal psychological and physiological conditions [4, 5]. These maternal conditions may affect FHR through the hormones transferred via the placenta or the changes in the oxygen and nutrition supply for the fetus. For example, a correlation was previously found between FHR and maternal stress and anxiety level [5, 6]. It was found to be linked to the transferred glucocorticoids and corticosteroid hormones through the placenta or released catecholamines, causing maternal vasoconstriction and limitation of the fetal oxygen and nutrient supply [5, 6]. Maternal relaxation was found to be associated with decreased FHR and increased variability [7]. In addition to the effect of maternal emotion, a significant correlation between the fetal and maternal diurnal heart rate rhythms was found with a phase lag of -2 to +2 hours, in a study by Lunshof et al. [8]. They hypothesized that the fetal suprachiasmatic nucleus, although not completely mature, is involved in transferring the maternal diurnal rhythm information to the fetal heart [8].

In addition to the relationship between maternal and fetal heart rates in large time scales discussed above, evidence of synchronization epochs between the heart rates at the beat-to-beat level was also reported [4, 9–12]. This short time coupling was found by Van Leeuwen et al., using phase synchronization analysis, as the phase locking of the rhythmic maternal and fetal heartbeats [9]. It was further shown by a model based approach, using the additive autoregressive processes with external contributing factors [10]. Fetal-maternal heart rate synchronization was further investigated in different settings, including controlled maternal respiration and maternal aerobic exercise [13, 14]. Results of those studies suggested that high maternal breathing rate may induce the synchronization as it occurred significantly more often at fast maternal breathing and less at slow respiratory rates [13]. Synchronization was found less often where mothers had exercised regularly, possibly due to an increased beat-to-beat differences, higher vagal tone and slower breathing rates [14]. As previously suggested, the short time fetal-maternal heart rate coupling might be via mechanical or auditory stimuli associated with the maternal rhythms, perceived by the fetus [10, 15]. However, the certain determination of the underlying mechanisms requires further investigation.

A factor which might contribute to the short time coupling of the maternal and fetal heart beats is the delay between them. For example, if the acoustic stimulation is assumed to be the reason behind the coupling, it is reasonable to consider the fetal auditory processing time, causing the latency of the fetal response. The latency of FHR changes in response to the Vibroacoustic Stimulation (VAS), maternal voice or displacement was reported in previous studies [16–18]. Therefore in the current paper, this time delay was investigated by analyzing the maternal-fetal heart rate coupling at different lags. The variation of the coupling strength and lag was further analyzed against gestational age, in order to assess the influence of fetal maturation.

Fetal behaviorial state may also affects FHR, particularly after 36 weeks of gestation, when the states can be identified by FHR analysis [19]. It may also have an influence on the maternal-fetal heart rate couplings. The behaviorial states are characterized by the simultaneous occurrence of specific FHR patterns, with or without eye and body movements and divided into: 1F (quiet sleep), 2F (active sleep), 3F (quiet awake), 4F (active awake) [20]. Although the states are commonly identified using long-term FHR monitoring and sonographic observation, they can also be classified based on the short-term FHR variability, such as RMSSD or SDNN, as reported by Lange et al. [21]. In this study we analyzed the effect of these parameters on the coupling for the fetuses in 32nd or later weeks of gestation.

Different from the previous studies on the fetal-maternal heart rate coupling, in this paper an information theory-based approach is used. We applied Transfer Entropy (TE) to investigate the interactions between fetal and maternal heart rates. TE is a non-parametric measure which can determine the coupling of two variables by quantifying the information transferred between them [22]. Using TE, we found the transfer of information between two variables on both directions, i.e. from maternal to fetal heart rate and vice versa. Without assuming any underlying model, TE can capture any linear and nonlinear link between the time series. Therefore it is more suitable than model-based measures such as Granger Causality (GC) for analyzing the physiological time series with nonlinear interactions [23]. TE has been used for investigating the coupling of physiological variables in various applications [24–26]. Improved methods and toolboxes for TE estimation have been recently proposed [27, 28].

## 2 Methods

### 2.1 Data

Maternal and abdominal ECG signals were recorded simultaneously from 65 pregnant women in Tohoku University Hospital. The pregnancies were all healthy, single and at the gestational age between 16 to 41 weeks. The cases were further divided into three age groups: early (16–25 weeks, 25 cases), mid (26–31 weeks, 18 cases) and late (32–41 weeks, 22 cases). ECG signals were recorded using 12 electrodes (10 electrodes on the mothers abdomen, one maternal reference electrode at the right thoracic position and one on the back). All signals were collected for 1 minute and sampled at 1 kHz with 16-bit resolution. Pregnant volunteers undergoing their routine prenatal tests had lain on the bed for five minutes before the one minute ECG measurement started. The study protocol was approved by Tohoku University Institutional Review Board and written informed consent was obtained from all participants.

### 2.2 Estimation of RR Intervals

Fetal ECG traces were extracted from the abdominal recordings by canceling the maternal ECG signal and separating by Blind Source Separation with Reference (BSSR) as described in our previous study [29]. A Pan and Tompkins-like QRS detector was used with refractory periods of 250 and 150 msec for detecting the maternal and fetal QRS, respectively; as proposed in previous studies [30, 31]. Maternal and fetal RR intervals (fRR and mRR) were then preprocessed by taking a moving window of five RR-intervals and replacing the middle sample by the average of the other four, if deviated by more than 20%. The fRRs and mRRs were resampled at 4 Hz, using cubic interpolation.

### 2.3 Transfer Entropy Analysis

The transfer entropy between two time series *X* = {*x*_1_, *x*_2_, …, *x*_*N*_} and *Y* = {*y*_1_, *y*_2_, …, *y*_*N*_} on *X* to *Y* direction, is calculated as:

$$TE_{X\to Y}=H\left(y_{i}|y_{i-t}^{l}\right)-H\left(y_{i}|y_{i-t}^{l},x_{i-\tau }^{k}\right)$$ (1)

$$=\sum_{y_{i},y_{i-t}^{l},x_{i-\tau }^{k}}p\left(y_{i},y_{i-t}^{l},x_{i-\tau }^{k}\right)log\left(\frac{p(y_{i}|y_{i-t}^{l},x_{i-\tau }^{k})}{p(y_{i}|y_{i-t}^{l})}\right)$$ (2)

where *i* is a given time point, *τ* and *t* are the time lags of *X* and *Y*, respectively, *k* and *l* are the lengths of the blocks containing the past values of *X* and *Y*, respectively. In this study TE was calculated for two directions: fetal to maternal (*F* → *M*) and maternal to fetal (*M* → *F*) heart rates. Therefore *X* and *Y* in the equations above denote fetal and maternal RR intervals after preprocessing and resampling. The conditional probabilities in Eq (2) are conditioned on $x_{i-\tau }^{k}=\left\{x_{i-\tau -k+1},x_{i-\tau -k+2},...,x_{i-\tau }\right\}$ and $y_{i-t}^{l}=\left\{y_{i-t-l+1},y_{i-t-l+2},...,y_{i-t}\right\}$. The transfer entropy is a non-negative measure of the reduction in uncertainty of *y*_*i*_ given $x_{i-\tau }^{k}$ and $y_{i-t}^{l}$, compared to given only $y_{i-t}^{l}$ [28].

As suggested in [28], due to the small sample size and computational reasons, the lag of the target and block lengths were all assumed to be one (*k* = *l* = *t* = 1). In this study 40 sample delays were considered for the source signal of TE, ranging from 250 msec to 10 sec in equal steps of 250 msec (= *T*_*sampling*_). The classic approach of fixed bins was used to estimate the probabilities in Eq (2), by allocating the data points to equally-spaced bins. Furthermore, RR-intervals were transformed by replacing them with their integer ranks sorted from smallest (1) to largest (*N*) values, to enhance the robustness of the measure against outliers and sparse regions of the distribution [28].

The same number of bins, *Q* = 10, was arbitrarily selected in each dimension which simplified the computation of TE as follows:

$$TE_{X\to Y}\left(\tau \right)\approx \sum_{a=1,b=1,c=1}^{Q}\frac{m_{a,b,c}}{P}log\frac{m_{a,b,c}m_{b}}{m_{b,c}m_{a,b}}$$ (3)

where *a*, *b*, and *c* are the index of bins along the transformed *y*_*i*_, *y*_*i*−1_, and *x*_*i*−*τ*_ time series, respectively, and *P* is the total number of triplets of transformed *y*_*i*_, *y*_*i*−1_, and *x*_*i*−*τ*_. Other bin numbers of *Q* = 6 and *Q* = 8 were also tested for comparison. The number of data points in the intersection of the one-dimensional bins are denoted by *m*_*a*, *b*, *c*_, *m*_*a*, *b*_, and *m*_*b*, *c*_, indexed by their subscript, and *m*_*b*_ is the number of data points at the *b* ^*th*^ bin in the transformed *y*_*i*−1_ dimension. More details on the computation of TE can be found in a previous study [28].

### 2.4 Surrogate Analysis

The significance of TE was statistically evaluated by surrogates using temporal shuffling of the time series as well as the Iterative Amplitude Adapted Fourier Transform (IAAFT) method. IAAFT surrogates retain the power spectrum and the amplitude distribution [32, 33]. In our experiment, TE was computed for 100 surrogates of the source time series (fRR or mRR for *F* → *M* or *M* → *F* directions, respectively). Given the TE from the original source was greater than the 95th percentile of the surrogate TE results, it was assumed to be significant. Only significant TE values were used for further analysis, e.g. mean TE was calculated over the delays at which TE was significant.

### 2.5 Maternal Respiratory Rate Estimation

Maternal respiratory rate was estimated through single lead ECG-Derived Respiration (EDR). Kernel Principal Component Analysis (K-PCA) technique was used, which was previously shown by Widjaja et al., to outperform PCA and R-peak amplitude methods in the extraction of the EDR [34]. The procedure of the EDR extraction can be found in [34] and summarized as follows. An input matrix *X* was formed by assembling *n* (no. of R-peaks) columns, each composed of a symmetric window of length *m* = 121 around each R-peak. Then K-PCA was applied to the input matrix, using Least Squares Support Vector Machines (LSSVM) toolbox LS-SVMlab v1.8 (http://www.esat.kuleuven.be/sista/lssvmlab/,Leuven, Belgium) [35]. Radial Basis Function (RBF) was used as a kernel with various parameter values ranging from $\sigma^{2}=0.1\sigma_{0}^{2}$ to $\sigma^{2}=100\sigma_{0}^{2}$, where $\sigma_{0}^{2}=m.\text{mean}\left(\text{var}\left(X\right)\right)$. The *σ* ^2^ value which resulted in the largest difference between the first and the sum of the remaining eigenvalues was selected. Using this value for kernel, the input data was reconstructed from the resulting first eigenvector in the feature space via preimage_rbf function of the LS-SVMlab toolbox. EDR was estimated as a row of this reconstructed observation.

The maternal respiratory rate was estimated from the EDR signal, using an algorithm proposed by Cysarz et al., summarized as follows [36]. EDR was resampled at 10 Hz using cubic spline interpolation. Then a band-pass filter was applied in the range of 0.1–0.45 Hz using a least-square FIR filter. The filtered signal was standardized by dividing by the 75 percentile of all detected local maxima, in order to exclude the influence of single oscillations with extreme amplitudes. The local maxima which exceeded 0.3 were further used and the average of the distances between the successive local maxima was calculated.

### 2.6 Statistical Analysis

The mean and maximum of (significant) TE as well as the lags resulting in maximum TE were all compared against different age groups by non-parametric statistical analysis. Mann Whitney Wilcoxon (MWW) test was used to compare maximum transfer entropy and corresponding delay for early, mid and late age groups. P-value of 0.05 was chosen as the level of significance. MWW test was also used for comparison of TE for different ranges of RMSSD and SDNN, each divided into two groups: high RMSSD ≥4 msec (6, 5 and 13 fetuses in early, mid and late gestation, respectively), low RMSSD <4 msec (19, 13 and 9 fetuses in early, mid and late gestation, respectively), high SDNN ≥12 msec (5, 8 and 15 fetuses in early, mid and late gestation, respectively), low SDNN <12 msec (20, 10 and 7 fetuses in early, mid and late gestation, respectively). The correlation of mean FHR, RMSSD and SDNN with TE on both directions were also tested. In each case linear partial correlation was evaluated while controlling for the gestational age. The correlation of maternal respiratory rate and mean TE was analyzed through Pearson’s correlation and MWW was also used to compare TE for various breathing ranges; i.e. lower than 14 bpm (18 subjects), between 14 and 16 bpm (21 subjects) and higher than 16 bpm (26 subjects).

## 3 Results

### 3.1 Results of surrogate analysis

**Fig 1:**
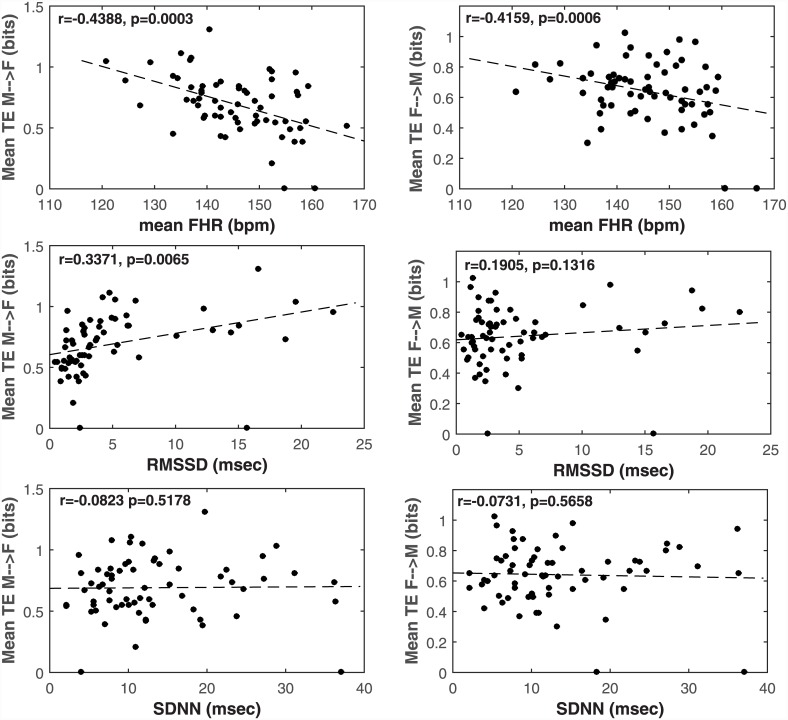
Regression plots of mean TE on both directions with mean, RMSSD and SDNN of FHR are shown. coefficient and p-values of partial correlation controlled for gestational age are also indicated. The cases shown with zero TE had insignificant TE according to the surrogate analysis by temporal shuffling.

TE was calculated at 40 lags and on two directions. Based on the surrogate analysis by temporal shuffling, TE was significant for 63 out of 65 cases, in each direction. On the other hand, TE was beyond 95% percentile of the surrogate TE generated by IAAFT in each direction for all the cases. The cases with insignificant (low) TE were excluded from further analysis. These insignificant cases were one early and one late gestation cases in maternal to fetal direction. *TE*_*F* → *M*_ was also not significant for that late gestation case, as well as another case in late gestation, while being significant for the rest of the cases. The fetuses with insignificant *TE*_*F* → *M*_ had the highest mean FHR (160.649 bpm and 166.736 bpm) among all 65 fetuses. The mean FHR for the fetuses with insignificant *TE*_*M* → *F*_ was also high, compared to other cases (160.649 bpm and 154.698 bpm). These cases are specified as *TE* = 0 in Fig 1, for comparison.

### 3.2 Comparison between gestational age groups

**Fig 2:**
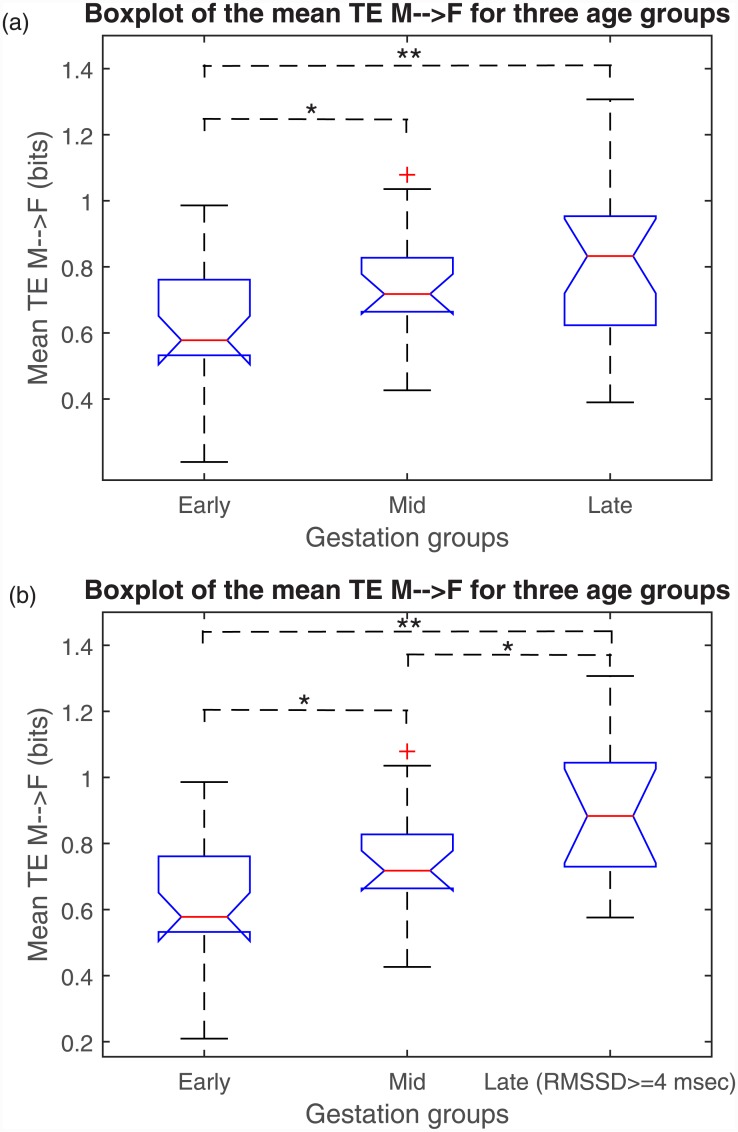
Comparison of the mean *TE*_*M* → *F*_ for different age groups is shown. Significant differences according to the pairwise comparison by MWW test with p-value <0.05 and p-value <0.01 are marked with (*) and (**), respectively. (a) Boxplot of mean *TE*_*M* → *F*_ for different age groups, (b)Boxplot of mean *TE*_*M* → *F*_ for different age groups, excluding the cases in late gestation group with RMSSD being smaller than 4 msec.

Both mean of and maximum of significant *TE*_*M* → *F*_ over the lags were significantly different for three age groups according to MWW test results (Table 1). Fig 2(a) shows the box plot of the mean *TE*_*M* → *F*_ for different age groups. According to MWW results as shown in Fig 2(a) and Table 1, both mean and maximum of *TE*_*M* → *F*_ significantly increased from early to mid and to late gestation, however no significant change was observed from mid to late gestation. This was further investigated by analyzing fetal heart rate variability as discussed in the following section. Mean and maximum of TE on the other direction (*F* → *M*) did not change significantly with gestation. However a decreasing trend was found in the mean *TE*_*F* → *M*_ with gestational progression, as it was negatively correlated with age (*r* = −0.393, *p* = 0.001 while controlling for the mean fHR).

**Table 1:** Results of Mann–Whitney—Wilcoxon test for changes of the estimated mean, maximum and delay of TE, as well as the maternal respiratory rate with gestational age.

	Early Gestation	Mid Gestation	Late Gestation
Mean *TE*_*M* → *F*_ (bits)	0.618±0.178 (A*,B**)	0.728±0.187 (A*)	0.808±0.227 (B**)
Max *TE*_*M* → *F*_ (bits)	0.698±0.196 (A*,B**)	0.816±0.201 (A*)	0.895±0.250 (B**)
Delay *TE*_*M* → *F*_ (sec)	4.490±2.844 (A*)	5.931±2.818 (A*,C*)	4.012±3.025 (C*)
Mean *TE*_*F* → *M*_ (bits)	0.673±0.170	0.687±0.160	0.626±0.156
Max *TE*_*F* → *M*_ (bits)	0.756±0.192	0.777±0.184	0.702±0.170
Delay *TE*_*F* → *M*_ (sec)	4.810±3.058	3.958±2.701	5.000±3.070
Maternal respiratory rate (bpm)	14.833±2.301 (B*)	15.218±2.074	15.904±2.587 (B*)

The mean ± Standard Error (SE) (msec) of the values for different age groups are shown. Significant differences between pairs of age groups: early vs mid, early vs late and mid vs late gestations are marked by (A), (B) and (C), respectively. The letters are also marked with (*) or (**) depending on the p-value of MWW test being <0.05 or <0.01, respectively.

Similar results were obtained when the number of bins was changed to *Q* = 8; an increase in *TE*_*M* → *F*_ was found with gestational age (KW *p* = 0.025), particularly from early to mid gestation (MWW *p* = 0.037), as well as a decreasing trend for *TE*_*F* → *M*_ from early to late gestation (nearly significant MWW *p* = 0.056). Similarly for *Q* = 6 bins, an increase was found in mean *TE*_*M* → *F*_ with gestational age (MWW *p* = 0.066, nearly significant for early to late gestation). Also a slight decrease in mean *TE*_*F* → *M*_ with age (MWW *p* = 0.081 from mid to late gestation) was observed and it was negatively correlated with age (*r* = −0.244, *p* = 0.051 while controlling for the mean fHR).

Delays which resulted in maximum TE were also analyzed for different gestation groups, as summarized in Table 1. The lag associated with maximum *TE*_*M* → *F*_ significantly decreased from mid to late gestation and increased from early to mid gestation. However the latter is not as valued as the former, considering the small value of *TE*_*M* → *F*_ at the early gestation. No significant change in delay was found on the other direction (*F* → *M*). By changing the number of bins from *Q* = 10 to *Q* = 6, similarly the delay decreased with age in both directions (e.g. MWW *p* = 0.05 for delay of *TE*_*M* → *F*_ for mid to late gestation, and for the delay of *TE*_*F* → *M*_ MWW *p* = 0.02 and *p* = 0.09 were found for early to late and mid to late gestation comparison, respectively). For the choice of *Q* = 8, the delay was also slightly decreased with gestational age in both directions, but it was not statistically significant.

### 3.3 Effect of short-term FHR variability

The correlation of mean TE was tested with mean, RMSSD and SDNN of FHR, all controlled for the gestational age. The regression plots with all correlation coefficients are shown in Fig 1. Mean *TE*_*M* → *F*_ was found only positively correlated with RMSSD (*r* = 0.337, *p* = 0.006) and negatively with mean FHR (*r* = −0.439, *p* < 0.001), while it was not significantly correlated with fetal SDNN (*r* = −0.082, *p* = 0.518). Considering that a significant increase in mean *TE*_*M* → *F*_ was found from early to mid, but not from mid to late gestation, we tested the changes in mean *TE*_*M* → *F*_ for the fetuses in late gestation with RMSSD ≥4 msec. As shown in Fig 2(b), for the fetuses with RMSSD ≥4 msec, there was a further increase from mid to late gestation (MWW p = 0.039), while no significant change for the fetuses with RMSSD <4. We also tested the relationship with the maternal heart rate variability features (mean heart rate, SDNN and RMSSD) and found no significant correlation. Finally, mean *TE*_*F* → *M*_ was only correlated significantly with mean FHR (*r* = −0.416, *p* = 0.001).

### 3.4 Effect of maternal respiration

No significant correlation was found between maternal breathing rate and mean *TE*_*M* → *F*_ or *TE*_*F* → *M*_, with or without controlling for fetal heart rate, RMSSD or age. MWW was also used to compare TE for various breathing ranges; lower than 14 bpm, between 14 and 16 bpm and higher than 16 bpm. According to MWW results, no significant difference was found between mean TE with different breathing rate ranges, over all or specific age groups and on any directions. Overall no relationship was found between the average maternal respiratory rate and *TE*_*M* → *F*_ or *TE*_*F* → *M*_.

## 4 Discussion

Assessing the responses of the fetuses to the stimuli in their environment, provides markers of their well-being and development. One of the common and easy-to-measure responses is the change in FHR. It was previously hypothesized that the fetus responds to mechanical or auditory stimuli associated with the maternal rhythms, which results in short time coupling between the fetal and maternal heart rates [10, 15]. Different from previous model based approaches, we used a non-parametric measure which could detect any linear and nonlinear relationship of two variables, based on the transferred information. Moreover using transfer entropy enables analysis of coupling in each separate direction. The significance of the coupling was statistically validated against surrogate pairs, and evidence of significant transfer of information was found on both directions.

The previous works in the literature reported transient coupling, by evaluating the coordination using phase synchronisation [9, 15]. There are two issues with that approach. Firstly, the interactions may be beyond the phase coordination and may not be detectable by phase synchronization for some uncoupling epochs, while the TE approach used in the current paper can capture any linear and nonlinear links without assuming any underlying model. Secondly, the phase synchronization approach only examined instantaneous coordination. By assuming a delay in the couplings, additional interactions were detected in our study, which would be missed by the previous approaches. Even if the coordination is transient, it would be manifested in TE results, since TE is calculated over the whole time series.

The results of this paper showed that the coupling from the mother to the fetus becomes stronger with advancing gestation. Particularly, the transfer entropy from the mother to the fetus significantly increases after 26 weeks of gestation compared to 16–25 weeks. This result is in agreement with the maturation process of the fetal response to the stimuli from the maternal rhythms, since both tactile and auditory systems become operational after 26 weeks of gestation [16]. It was previously reported that the maturation of human fetal response (in form of changes in FHR) to Vibroacoustic Stimulation (VAS) starts at around 26 weeks and reaches maturity at about 32 weeks [16]. Our result is also in line with the increasing sympathetic activity with gestational progression, alongside the development of fetal Autonomic Nervous System (ANS) and its function in regulation of FHR [37]. Development of the sympathetic control during mid gestation is also concomitant to the nonlinear heart period dynamics, which is suggested to be involved with sympathetic regulation and also possibly the sympatho-vagal interactions [38]. The nonlinearity of the FHR dynamics particularly in mid and late gestation was also our motivation for using TE. With advancing gestation particularly around mid gestation, the fetus receives more information from the mother and reacts better, while the FHR shows more complexity and nonlinear dynamics. Therefore, the transfer entropy from the mother to the fetus may provide a marker to assess the development of fetal sensory and autonomic nervous systems.

Although we found an increase in *TE*_*M* → *F*_ from early (16–25 weeks) to mid (26–31 weeks) gestation, no significant increase was found from mid to late (32–41) gestation. A factor which might be involved in the coupling in late gestation, is the fetal behavioral state. A higher FHR variability is generally observed in active periods after 28th week, and the states can be identified by FHR analysis after 36 weeks of gestation [19]. As previously reported by Lange et al., the fetal behavioral states can be classified based on the differences in short-term FHR variability such as RMSSD or SDNN [21]. In this study a positive correlation was found between RMSSD and *TE*_*M* → *F*_, and there was a further increase in *TE*_*M* → *F*_ from mid to late gestation for the fetuses with RMSSD ≥4 msec, which is associated with active state of the fetus [21]. However, a thorough assessment of the fetal behavioral state can be better performed through long-term FHR monitoring and sonographic observation, which is suggested for future studies.

The fetus also provides feedback to maternal systems throughout its own development, which can evoke a maternal physiological response. Our results showed that the transfer of information from fetal to maternal heart rate was negatively correlated with age. The decrease in *TE*_*F* → *M*_ with gestational progression is possibly because the fetus requires less from the mother when most organs are developed towards delivery. The mature fetuses have also more stable and developed ANS in the late gestation. TE on both directions was negatively correlated with mean FHR and the cases with insignificant TE had higher FHR compared to other fetuses. Although it is not possible to comment on the causal link between FHR and TE, but this negative correlation implies that at high FHR it may be difficult for the fetus to maintain the link with the maternal heart rate.

In this study the coupling between maternal and fetal heart rate was observed at various delays. These lags may reflect the fetal (e.g. auditory or tactile) processing time before responding to the stimuli from mother. Similarly, a latency was previously found for FHR changes in response to the VAS, maternal voice or displacement [16–18]. The lags for the short-term relationship between maternal and fetal heart rates were also considered in a model-based analysis by Riedl et al. [10]. They found a short-term synchronization through which, the maternal beats described the FHR fluctuations as a predecessor with a lag of 4 to 5 fetal beats. Considering that the fetuses in their study were at 34 to 40 weeks of gestation, their finding is consistent with our results for the late gestation group. We further included the delays up to 10 sec (around 20–28 fetal beats) in our analysis to allow for detection of longer delays particularly for the fetuses in earlier gestation. In addition, the changes of the lag corresponding to the maximum TE was analyzed for different gestational ages. The lag for *TE*_*M* → *F*_ was significantly shorter in the late gestation. This is an evidence of faster fetal sensory processing time and shorter latency of fetal response to the stimuli from maternal rhythms, according to the maturation of sensory systems and ANS. Previous studies observed similar results for the late gestation stage, such as decreased latency of fetal response to maternal voice [17].

Previous studies tested the relationship between fetal and maternal heart rates in controlled maternal respiration setting and suggested that the relationship may be induced by high maternal respiratory rates [13]. They found that the synchronization occurred significantly more often at fast maternal breathing and less at slow respiratory rates [13, 15]. In this study we tested TE for average maternal respiratory rate, derived from maternal ECG. No significant relationship was found between maternal respiratory rate and *TE*_*M* → *F*_ or *TE*_*M* → *F*_. Our analysis was only based on the average respiratory rate for each mother. An accurate breath-by-breath measurement of maternal respiration is suggested for future studies, to be considered as a confounding variable in TE analysis. Therefore, it would be possible to evaluate the causal effect between maternal-fetal heart rates, conditioned on the maternal respiration.

In this study transfer entropy was evaluated based on one-minute recordings, which is the standard fetal ECG measurement protocol to minimize the inconvenience for the participating mothers. For future studies, it is recommended to use longer recordings to investigate the effect of sample size and study the changes in information transfer over time for each fetus. Furthermore, application of other methods for measuring the coupling, for example to improve the quantification of coupling for small sample sizes [39], is recommended for future investigations. Longer recordings also provide the analysis of nonstationarity in FHR, as a factor with possible influence on TE analysis. Nonstationarity of FHR becomes more pronounced in the late gestation due to the fetal movement. An accurate evaluation of the non-stationarity in the FHR requires longer recordings, e.g. to measure the inconsistency of baselines using acceleretion/deceleration patterns [40]. Analysis of the nonstationarity and its influence on TE measures are left for the futures studies.

## 5 Conclusion

Using transfer entropy as a non-parametric measure, significant couplings were found between maternal and fetal heart rates on both directions for 63 of 65 fetuses. Maternal to fetal TE increased from early to mid gestation, along maturation of fetal ANS and sensory (e.g. auditory and tactile) systems. It was further increased from mid to late gestation, except for the fetuses with low RMSSD (<4msec) of heart rate, possibly due to their quiet sleep state. The fetal to maternal TE was negatively correlated with gestational age, showing a decrease in the feedback from fetus to the mother towards delivery. Furthermore, the delay at which maximum information transferred from mother to the fetus was shorter in the late gestation, implying the short fetal processing time and latency in responding to the stimuli from the mother. Results suggest that the assessment of the coupling strength and latency throughout gestation can provide clinical markers of healthy versus pathological fetal development.
